# Breaking new ground: penetrating trauma and cisterna chyli injury: a case report

**DOI:** 10.1093/jscr/rjaf093

**Published:** 2025-03-05

**Authors:** Nawaf AlShahwan, Saleh Husam Aldeligan, Mohammed Basem Bayari, Ahmed Alburakan, Hassan Mashbari, Thamer Nouh

**Affiliations:** Trauma and Acute Care Surgery Unit, Department of Surgery, College of Medicine, King Saud University, PO Box 2925, Riyadh 11461, Saudi Arabia; College of Medicine, King Saud University, PO Box 2925, Riyadh 11461, Saudi Arabia; College of Medicine, King Saud University, PO Box 2925, Riyadh 11461, Saudi Arabia; Trauma and Acute Care Surgery Unit, Department of Surgery, College of Medicine, King Saud University, PO Box 2925, Riyadh 11461, Saudi Arabia; Department of Surgery, Faculty of Medicine, Jazan University, PO Box 6809, Jazan 82817-28204, Saudi Arabia; Trauma and Acute Care Surgery Unit, Department of Surgery, College of Medicine, King Saud University, PO Box 2925, Riyadh 11461, Saudi Arabia

**Keywords:** chyle leak, traumatic injury, penetrating trauma, cisterna chyli, trauma surgery

## Abstract

Here we discuss a rare case of cisterna chyli injury resulting from penetrating trauma, with no prior reported cases in the literature related to such injuries caused by penetrating trauma. The patient, a 21-year-old male, presented with multiple stab wounds, prompting exploratory laparotomy. Penetrating wounds to the stomach and a pancreatic laceration with retroperitoneal hematoma were identified. Notably, chyle leakage from the cisterna chyli was managed by clipping lymphatic branches. The paper emphasizes the importance of intraoperative identification and control of chyle leaks, as they can impact nutritional status and wound healing. Various maneuvers, including intraoperative indocyanine green lymphangiography and postoperative monitoring of drain output, are discussed for detecting and managing chyle leaks. The patient's postoperative course was uneventful, highlighting successful management and discharge on Day 11 in good health.

## Introduction

Trauma to the cisterna chyli, chyle may leak into the peritoneum either through lymphatic fistula or via back pressure on the intestinal lymphatics. The development of abdominal symptoms following chylous leakage generally takes days to weeks unless there is an associated mesenteric tear [1]. Penetrating injuries to the anterior abdominal wall have several approaches to manage them [2]. Laparoscopic examination to identify fascial penetration is an option in patients who have normal vitals and are unable to undergo reliable serial abdominal exams due to intubation or intoxication, once a peritoneal violation is identified then the patients usually undergo an exploratory laparotomy [3]. Intra-abdominal extravasation of chyle is a rare sequelae of blunt trauma. Chylous ascites may result from rupture of the thoracic duct, the cisterna chyli, or abdominal lymphatics [4]. The thoracic duct or cisterna chyli may be ligated without harmful sequelae due to abundant collaterals. Total parenteral nutrition may be of value to temporarily decrease abdominal lymphatic flow [5].

## Case report

A 21-year-old male presented to the emergency department with multiple stab wounds including bilateral mid thighs, left hand, right facial area and anterior abdominal wall in the epigastrium 3–4 cm above the umbilicus. Upon arrival the patient was agitated and combative, he had a Glascow Coma Scale 13/15, a blood pressure 103/61, and a pulse of 107. He was immediately intubated for airway protection and to be able to perform an evaluation. Imaging (X-rays and E-FAST) were negative. There was no vascular compromise in all four limbs so the patient was shifted to the operating room for exploration to investigate whether the peritoneum was violated or not. Diagnostic laparoscopy revealed peritoneal violation so we converted to an exploratory laparotomy. The exploratory laparotomy revealed penetrating wounds to the stomach (both anterior and posterior walls), this was managed with simple suture primary repair using PDS. We then identified a pancreatic laceration in the lower part of the pancreas with an associated retroperitoneal hematoma. Further exploration of the injury on the lower border of the pancreas showed no active bleeding from the major vessels, and we noted some white colored fluid that was draining from the retroperitoneum. Further examination this was deemed to be chyle and that was managed with clipping the lymphatic branches. There were no other injuries identified in the rest of the abdomen so we proceeded to place a drain underneath the pancreas and closed the abdomen. Postoperative course was uneventful, as the patient was not passing gas on Day 5 and was found to be distended on physical examination, so he underwent a CT scan with oral contrast that showed signs of ileus with dilated small bowel loops and no leak from the gastric repair in addition to that no intraabdominal collections were identified. The drain was removed after the patient tolerated a regular diet with no output from the drain. He was discharged on Day 11 post injury in excellent health.

## Discussion

We report this case since there were no reported cases in the literature of cisterna chyli injury related to penetrating trauma. A few reports published were secondary to blunt trauma. Chyle leak can adversely affect the nutritional status of the patient and wound healing. It results in a significant loss of proteins and fats, induces severe inflammatory reaction due to inflammatory cytokines, can macerate the surrounding structures. The increased pressure due to accumulation of chyle can cause intra-abdominal collections and wound dehiscence. [6] It is important to identify chyle leak intra-operatively and control it with ligation or clipping. However, minor leaks may be difficult to identify during surgery in certain areas of the body such as the neck due to branching of the thoracic duct before draining into the venous system. Some of the described maneuvers in the literature are utilizing the trendelenburg position or applying pressure over the abdomen are various maneuvers that can help identify the chyle leak intra-operatively when operating in the neck [7]. In the abdomen or chest, chyle leak could be identified through real-time intraoperative indocyanine green lymphangiography as imaging as described by Das *et al*. [8]. Described by Cheng *et al*. [9] also, postoperatively, chyle leak could be identified by nuclear There are multiple ways to approach chyle leak. It mainly depends on the patient status and etiology of the leak [10]. Early on, the underlying cause of the leak should be identified and then treated. In our case, the cause was a penetrating traumatic injury managed by clipping the cisterna chyli. However, the most common cause of chyle leak is malignancy; other etiologies include inflammatory diseases, infectious process, congenital malformations, and cirrhosis. Chyle leaks vary with multiple causes such as the leak severity, nutritional and hydration status, local inflammation, immunological status of the patient, and local expertise. Usually, we start conservatively by Nill Per Os and total parenteral nutrition in the first postoperative period and then we introduce a high protein and low-fat diet to avoid chyle generation with stool softeners and elevation of head of the bed [11]. Here we describe a rare case of a penetrating traumatic injury of cisterna chyli that, to our knowledge, was never reported in the literature. Cisterna chyli in our patient case was injured through an anterior stab wound that traversed through both anterior and posterior walls of the stomach and injured the cisterna chyli. Luckily, the patient did not have an injury to the inferior vena cava or aorta. We managed the patient through an exploratory laparotomy repairing his stomach primarily and clipping the cisterna chyli proximally and distally to prevent leakage in addition to leaving drains. Postoperative conservative considerations were diet adjustments including high protein, free medium chain triglycerides, and low sodium intake, in addition to drain output monitoring that was insignificant until the patient was discharged from the hospital.
